# Assessment of the Short-Term Effects after High-Inductive Electromagnetic Stimulation of Pelvic Floor Muscles: A Randomized, Sham-Controlled Study

**DOI:** 10.3390/jcm9030874

**Published:** 2020-03-23

**Authors:** Kuba Ptaszkowski, Bartosz Malkiewicz, Romuald Zdrojowy, Lucyna Ptaszkowska, Malgorzata Paprocka-Borowicz

**Affiliations:** 1 Department of Clinical Biomechanics and Physiotherapy in Motor System Disorders, Faculty of Health Science, Wroclaw Medical University, Grunwaldzka 2, 50-355 Wroclaw, Poland; kuba.ptaszkowski@umed.wroc.pl (K.P.); malgorzata.paprocka-borowicz@umed.wroc.pl (M.P.-B.); 2 Department and Clinic of Urology, Faculty of Medicine, Wroclaw Medical University, Borowska 213, 50-556 Wroclaw, Poland; bartosz.malkiewicz@umed.wroc.pl (B.M.); romuald.zdrojowy@umed.wroc.pl (R.Z.); 3 Department of Physiotherapy, Opole Medical School, Katowicka 68, 45-060 Opole, Poland

**Keywords:** electromagnetic field, pelvic floor muscle, urinary incontinence

## Abstract

Background: Physiotherapy should be performed by patients with stress or mixed urinary incontinence (SUI and MUI) to increase the strength and endurance of the pelvic floor muscles (PFMs). A method that can positively affect the pelvic floor is stimulation with high-inductive electromagnetic stimulation (HIES). The aim of the study was to evaluate the PFMs after the application of HIES in women with SUI and MUI by using surface electromyography (sEMG). Methods: This was a prospective, randomized, single-blind study with a sham intervention group. The participants were randomly assigned to the HIES group or sham group. The outcomes were features of the bioelectrical PFM activity assessed using sEMG and endovaginal probes. A single-session intervention in the HIES group included 20 min of HIES with an electromagnetic induction intensity of 2.5 T. Results: In the HIES group, there was a statistically significant difference in the PFM sEMG activity during “contractions” (*p* < 0.001) and “quick flicks” (*p* = 0.005). In the intergroup comparison, higher PFM sEMG activity after the intervention (“contraction”) was observed in the HIES group than in the sham group (after: *p* = 0.047; 1 h after: *p* = 0.017). Conclusions: The assessed HIES method seems effective for SUI and MUI patients in the short term and shows an advantage over the sham intervention in the assessment of PFM contractions.

## 1. Introduction

In women, weakening of fascia–ligament–muscle structures of the pelvic floor and disorders of the blood supply to the tissues of this area often cause symptoms of stress or mixed urinary incontinence (SUI and MUI) [1,2,3,4,5]. Physiotherapy (including physical therapy) should be performed by patients with these types of symptoms to increase the strength and endurance of the pelvic floor muscles (PFMs) as well as increase the elasticity of the pelvic floor structures [6,7,8,9,10]. It seems that one of the methods of physical therapy that may positively affect the pelvic floor is stimulation with high-inductive electromagnetic stimulation (HIES; high-inductive, deep-penetrating, pulsed electromagnetic stimulation) [11,12,13,14]. The use of other physical therapy in the treatment of SUI has been described in the literature [15,16,17,18], and electrotherapy is mainly used [16,17,18]. Many authors [9,10,19,20] have indicated the need for searching for new, more effective methods that can reduce the occurrence of urinary incontinence symptoms. Among the electrotherapeutic methods, laser stimulation [21,22] and magnetic stimulation therapy [11,23,24,25,26,27,28] have been mentioned. The trend until now in the treatment of urinary incontinence has been to use a magnetic field with a frequency of 0.5 to 50 Hz and a magnetic induction intensity of 0.1 to 20 millitesla (mT) [24,25,28]. In the present project, the use of HIES with a frequency from 1 to 50 Hz has been proposed, and the maximal magnetic induction intensity amounts to 2.5 tesla (T). The new electromagnetic stimulation notably differs from perceptible tingling and tissue vibration during the treatment. Manufacturers recommend HIES as an effective, comprehensive, noninvasive and safe method for the treatment of SUI [13,14,29]. Its effects on peripheral nerve stimulation, muscle activation and effectiveness in improving the composition of collagen structures and blood circulation have been highlighted [12,13,14].

The impact of stimulation with HIES (a single-session intervention) on the pelvic floor structures in women with SUI or MUI is the foundation of this research project, as there is evidence that it can influence the tissue-healing process, improve neural tissue regeneration, increase elasticity and tissue perfusion and alleviate pain in people with chronic conditions [12,13,14,24,25]. The lack of randomized studies with a sham intervention on the impact of HIES on the pelvic floor muscles prompts this type of study [13,14]. 

The primary aim of the study was to objectively evaluate the PFMs after the application of HIES of the pelvic floor in women with SUI and MUI symptoms by using electromyography. The main hypothesis is that the HIES intervention increases the resting and functional bioelectrical activity of the PFMs. 

## 2. Methods

This was a prospective, randomized, single-blind study with a sham group evaluating the persistent changes in specific parameters after HIES. The study was carried out at the Clinic of Urology and Urologic Oncology at the University Hospital in Wroclaw, Poland, between January 2017 and December 2019. The study protocol was approved by the Bioethics Committee of Wroclaw Medical University (KB-97/2017), and the study was conducted in accordance with the ethical principles of the Declaration of Helsinki. All patients provided written and informed consent.

Patients were recruited from the urological and gynecological outpatient clinic of the University Hospital in Wroclaw (Poland). The target group of the study included women with symptoms of SUI and MUI. All recruited participants were screened according to the inclusion and exclusion criteria to determine their eligibility for the study. Inclusion criteria were (1) provision of informed consent to participate in the study; (2) obtaining permission to participate in the study based on the assessment of all the inclusion and exclusion criteria by the members of the research team (urologist, physiotherapist); (3) lack of contraindications for the sEMG measurement; (4) lack of contraindications for HIES; and (5) occurrence of symptoms of SUI or MUI (for at least 1 year). The exclusion criteria were as follows: (1) a history of gynecological surgeries; (2) a history of surgeries within the abdomen, pelvis or lower limbs in the last 10 years; (3) on the day of examination, the occurrence of injuries of the lower limb, pelvis or spine; (4) organ prolapse; and (5) third-degree urinary or fecal incontinence [11,17,30,31]. In addition, individuals with (6) any neurological symptoms; (7) systemic diseases; (8) diabetes mellitus; (9) lumbar or pelvic pain in the last 6 months were excluded [9,10,30]. Participants were also excluded if they had (10) any contraindications for HIES (pregnancy, cancer, heart pacemaker, hearing aid or other non-removable electronic devices, bleeding of any origin, pain of unknown origin, neurological diseases, epilepsy, very low blood pressure, metal pieces within the area of HIES application, radiotherapy or chemotherapy, malaise during the examination or infection of the urogenital tract) [13,14,29]; (11) allergy to nickel [9,30,32]; (12) the occurrence of pain during the study [9,30,32]; or (13) resignation during the study [9,30,32].

The participants were randomly assigned to one of two comparison groups (HIES group and sham group). Randomization was carried out using computer-generated random numbers (simple randomization). The participants were randomly assigned to groups in a 1:1 ratio. In the HIES group, HIES was applied, and the sham group was the control group (sham of HIES). To analyze the persistent changes in particular parameters, the sEMG activity was recorded immediately before (baseline), immediately after, and 1 h after the stimulation (1 h follow-up (FU)). The primary outcomes were features of the bioelectrical PFM activity, which was measured using sEMG and endovaginal probes. 

The protocol of the participant examination was as follows: consent to participate in the research was obtained; a medical interview with a clinical assessment of SUI/MUI symptoms using the International Consultation on Incontinence Questionnaire–Urinary Incontinence Short Form (ICIQ-UI-SF) [33] was conducted; instruction on the purpose of the measurements and testing procedures was provided; the patient was prepared for the measurements and appropriate intervention; sEMG measurements of the PFMs were taken with the patient in the supine position; and the appropriate intervention based on the group assignment was administered. The measurements were repeated immediately after the intervention and 1 h after the intervention.

**Figure 1:**
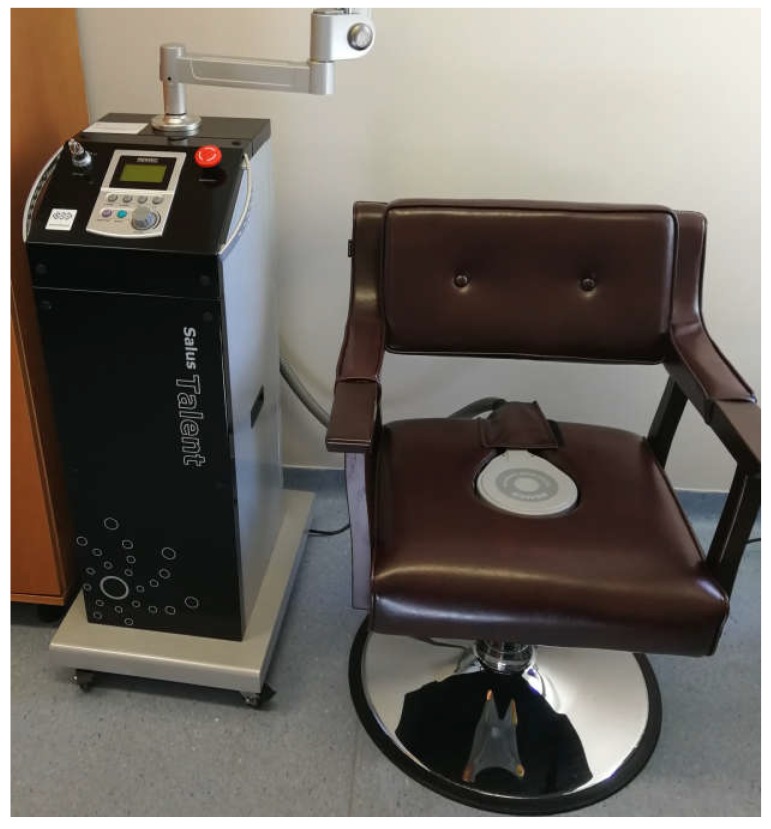
The device used for carrying out the intervention with high-inductive electromagnetic stimulation (HIES).

The intervention (a single-session intervention) in the HIES group included local 20 min HIES with an electromagnetic induction intensity of 2.5 T [13,14,29]. The SOLUS TALEN device (REMED, Daejeon, South Korea, Figure 1) was used to conduct HIES. For the stimulation, the manually set mode recommended by the manufacturer for individuals with symptoms of SUI and MUI was used (frequency of 10–50 Hz, pulse duration 3 s, pause time 6 s, magnetic induction of 2.5 T). The intervention was carried out in a comfortable and safe sitting position on a specialized chair with the special generator placed inside the seat. In the sham group, to exclude the effect of the impact of this device on the tissue, a specialized overlay on the generator was applied, which prevents the penetration of electromagnetic waves into the structures of the pelvic floor. The participants were not aware of which intervention they were receiving (single-blinding).

sEMG measurements were taken with an eight-channeled electromyograph MyoSystem 1400 L (Noraxon, Scottsdale, AZ, USA) and compatible endovaginal electrodes (a pear-shaped endovaginal electrode, Life-Care Vaginal Probe PR-02; Everyway Medical Instruments, New Taipei City, Taiwan). The sEMG recording frequency was set to the range of 10 to 450 Hz. The cut-off frequency for the high-pass filter in the amplifier was 10 Hz, and the cut-off frequency for the low-pass filter was 500 Hz. The level of common-mode rejection amounted to a minimum of 100 dB, and the input impedance for the EMG channels was higher than 100 MOhm. The system had high sensitivity in recording EMG signals (1 µV) [30].

For all measurements of PFM activity, the following testing conditions were used: “rest tone (initial)” (10 s of PFM activity at rest before functional measurements), “contractions” (5 × 10 s contractions, in which the participants tried to contract the PFMs and hold for 10 s), “quick flicks” (10 s measurements, in which participants performed short, quick contractions of the PFMs), “static hold” (in which the participants attempted to hold the PFM contractions for 60 s), and “rest tone (last)” (10 s of the PFMs at rest after the functional measurements).

The electromyographic signals were subjected to standard post hoc processing. They were rectified and smoothed using the root-mean-square (RMS) algorithm, and to reduce the phase shift, they were subjected to filtering. A narrow band-pass filter with a frequency range of 50 to 1000 Hz was used (finite impulse response filter—FIR filter). The results are presented in microvolts (µV).

Statistical analysis was performed using Statistica 13 (TIBCO Software Inc., Palo Alto, CA, USA). The required sample size was estimated based on the data from a pilot study (non-published data). Means and standard deviations of functional sEMG activity of the PFMs before and after HIES intervention were used in the analysis to estimate the sample size. The sample size was estimated for a two-sample paired-means test (paired *t* test) with the following parameters: a mean of 8.1 µV before the intervention; a mean of 10.4 µV after the intervention; a standard deviation of 2.4 µV; and a null difference of 0 µV. The alpha level was set at 0.05, and the power of the test was set at 0.8. It was also assumed that there were no correlations among the evaluated variables, and a two-sided null hypothesis was adopted. On the basis of the parameters, the estimated sample size corresponded to 18 women in each group. In addition, a 10% risk of losing patients in the follow-up assessment was assumed. The final sample size corresponded to 20 participants in each group. 

For the measurable variables, the median and quartiles were calculated. All tested quantitative variables were tested with the Shapiro–Wilk test to determine the type of distribution. The reliability and repeatability of measurements for sEMG activity were assessed using an intraclass correlation coefficient (ICC; in each case, *r* ≥ 0.90). Comparison of results between the groups was performed using the nonparametric U Mann–Whitney test or chi-squared test. A comparison of the intragroup results between consecutive measurements (baseline, after the intervention, 1 h FU) was carried out using the Friedman test with post hoc testing (Dunn test). For all comparisons, the level of *α* = 0.05 was assumed.

## 3. Results

**Figure 2:**
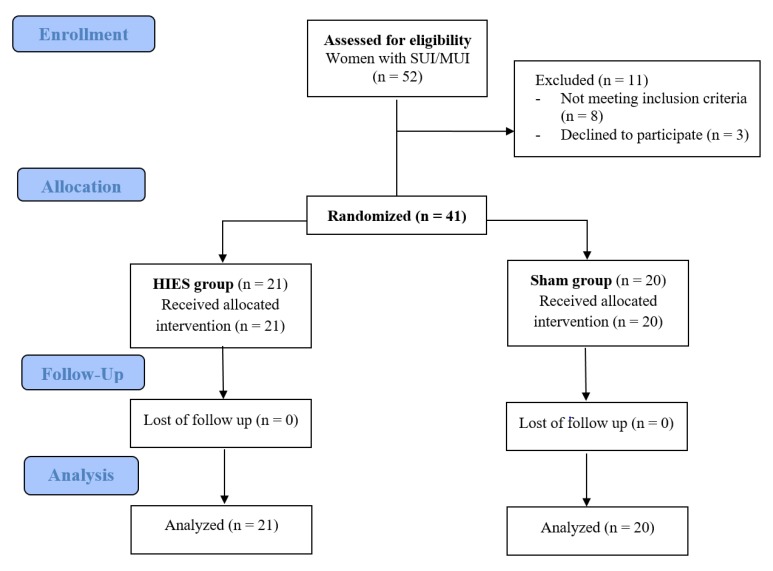
The CONSORT 2010 flow chart of patients in the study.

Fifty-two patients were eligible for the study. Based on the inclusion and exclusion criteria for the study, 41 patients took part in the measurements. Figure 2 presents the flow of the patients at each stage of the project. The participants’ demographic and clinical characteristics are shown in Table 1. There were 21 women aged 57–75 years (median = 64 years) in the HIES group and 20 women aged 57–77 years (median = 50 years) in the sham group. There were no significant intergroup differences in the patients’ characteristics.

In the HIES group, there was a significant difference in the sEMG measurements of PFM activity during “contractions” and “quick flicks” (Table 2). In the “contractions” after the intervention and at the 1-h FU, the PFM activity was higher by almost 2 µV (main effect—*p* < 0.001; post hoc test: baseline vs. after—*p* = 0.008; baseline vs. 1-h FU—*p* = 0.002; after vs. 1-h—*p* = 1.00). A similar result was recorded in the measurement of the “quick flicks” (main effect—*p* = 0.005; post hoc test: baseline vs. after—*p* = 0.14; baseline vs. 1-h FU—*p* = 0.049; after vs. 1-h—*p* = 1.00). In the sham group and in other measurements, no statistically significant differences were noted.

In the intergroup comparison, the PFM activity measured by sEMG after the intervention (“contractions”) was observed to be higher in the HIES group than in the sham group (Table 2). 

## 4. Discussion

This is the first randomized trial with a sham group that has been conducted to evaluate the effect of HIES on PFMs. In comparison with the sham group, the intervention group only showed higher signals during “contractions”. This result may be important for conducting additional research using this intervention.

Two studies conducted in 2019 used HIES [13,14]. In these publications, the term “high-intensity focused electromagnetic field” (HIFEM) is used, but it refers to the same electromagnetic field with 2.5 T inductance that was used in this study. In the studies by Samuels et al. [14], the authors evaluated the safety and efficacy of HIFEM for the treatment of urinary incontinence with an emphasis on its effects on prospective patients’ quality of life. The researchers noted that after the sixth session, 61 out of 75 patients (81.33%) reported a significant reduction in their symptoms. They conclude that HIFEM technology can be used to safely and effectively treat many patients suffering from urinary incontinence [14]. A team of researchers led by Silantyeva [13] assessed the immediate efficiency of HIFEM therapy and electrostimulation for the treatment of weakened PFMs accompanied by UI. In their opinion, the post-treatment results suggest that HIFEM technology is suitable for the treatment of PFM weakening and is more effective than electrostimulation in the short term. The researchers recommend HIFEM as a treatment option for weakened PFMs and UI [13].

Samuels et al. [14] and Silantyeva et al. [13] showed the positive effects of using a high-inductive electromagnetic field, which correlate with the results in this study. In the above studies, randomization and a sham group were not used, which may constitute a limitation of those studies. However, the conclusions of this study, as well as those described above, provide grounds for using a high-induction electromagnetic field therapeutically for the treatment of UI symptoms.

Although there is limited research on HIES in the literature, we identified a study on the use of a low-inductive magnetic field (magnetic stimulation therapy) in patients with SUI, which also suggests the effectiveness of HIES. By searching the literature, we identified several studies [24,25,27,28,34,35,36,37,38,39,40,41,42] of differing and often low scientific quality showing the influence of a pulsed magnetic field on the PFMs. Several reports [24,25,28,35,36,38,39] describe the use of the same therapeutic apparatus (NeoControl chair, Kitalpha Med Ltd., Germany), which generates a pulsed magnetic field (low-inductive). In all of these cases, stimulation for 20 min was applied (10 min, 5–10 Hz; 10 min, 50 Hz). These works mainly address the effectiveness of a low-frequency magnetic field in increasing PFM strength, alleviating symptoms of SUI and reducing the subjective perception of pain associated with insufficiency of the pelvic floor. Moreover, the authors [25,35,39] emphasize the need for additional studies that are well-designed.

Currently, therapy with an electromagnetic high-induction intensity of 2.5 T (HIES) is of great interest and may be more important than that with a low-inductive magnetic field. This may be due to the fact that muscle contraction is noticeable, which is caused by the generation of a high-inductive, deep-penetrating, pulsed electromagnetic field [13,14,29].

In this study, in the HIES group after the intervention, we observed an increase in sEMG activity of PFM during “Contraction” and “Quick flicks”. An increase in sEMG of PFM activity may contribute to the reduction of SUI- or MUI-related symptoms. Studies of Dornowski et al. [42] show that after the PFM exercises, there was an increase in sEMG activity with a decrease in urine loss. Similar results were obtained by Alves et al. [43]: PFM training program reduced SUI symptoms that correlated with increased sEMG activity. The use of HIES seems justified in the treatment of symptoms associated with SUI and MUI, taking into account the fact that the activity of sEMG of PFM increases after using one-session HIES intervention.

The limitations of this work are the presentation of short-term results and the use of a one-session intervention. This study serves as a foundation for future studies in which several recommended therapeutic sessions for patients with urinary incontinence are administered. Another limitation of the work is the fact that the participants had different levels of pelvic floor damage. In future studies, additional pelvic floor structure assessment methods are needed.

## 5. Conclusions

The assessed HIES method seems effective for SUI and MUI patients in the short term and shows an advantage over the sham intervention in the assessment of PFM contractions. The use of this intervention significantly increases the functional bioelectrical activity of PFMs. To assess clinical parameters in the long term, additional studies are required.

## Figures and Tables

**Table 1:** Urinary incontinence patients’ demographic and clinical characteristics in the HIES group and sham group.

Quantitative Variables		HIES Group (*n* = 21)		Sham Group (*n* = 20)		*p*-Value ^1^
		Me	Q1–Q3	Me	Q1–Q3	
Age (years)		53	44–56	50	38–59	0.13
Weight (kg)		64	61–69	64	57–73	0.91
Height (m)		1.64	1.63–1.66	1.66	1.64–1.70	0.26
BMI (kg/m^2^)		24	22–26	24	22–26	0.84
Occurrence of urinary incontinence symptoms (years)		8	5–10	5	2–7	0.052
ICIQ-UI SF score		6	5–8	8	4–10	0.51
**Qualitative Variables**		***n***	**%**	***n***	**%**	***p*-Value ^2^**
Kind of work	Physical	2	10	3	15	0.86
	Mental	11	52	10	50	
	Physical/Mental	8	38	7	35	
Number of deliveries	0	2	10	2	10	0.97
	1	4	19	5	25	
	2	13	62	11	55	
	3	2	10	2	10	

^1^ U Mann–Whitney test; ^2^ chi-squared test; HIES: high-inductive electromagnetic stimulation; *n:* number of participants, Me: median; Q1: first quartile; Q3: third quartile; %: percent; BMI: body mass index; ICIQ-UISF: International Consultation on Incontinence Questionnaire–Urinary Incontinence Short Form.

**Table 2:** Intergroup and intergroup comparison of the sEMG results for the pelvic floor muscle (PFM) in the HIES and sham groups of patients.

sEMG Activity of PFM (µV)	Measurement	HIES Group		Sham Group		*p*-Value ^1^
		Me	Q1–Q3	Me	Q1–Q3	
Rest Tone (initial)	Baseline	3.9	3.1–5.1	3.3	2.8–4.1	0.20
	After	4.3	3.3–5.0	2.9	2.6–4.4	0.08
	1-h FU	4.1	3.5–4.9	3.3	2.4–4.8	0.10
*p*-value ^2^		0.49		0.79		
Contraction	Baseline	10.1	8.3–12.3	9.4	7.5–11.7	0.54
	After	11.7	9.7–13.8	10.5	8.3–12.1	**0.047**
	1-h FU	12.0	10.1–13.9	11.0	8.0–12.0	**0.017**
*p*-value ^2^		**<0.001**		0.89		
Quick flicks	Baseline	10.6	8.5–13.5	10.5	8.1–12.1	0.82
	After	12.1	9.7–14.9	10.0	8.8–12.9	0.20
	1-h FU	12.4	10.1–15.3	11.1	8.6–12.0	0.08
*p*-value ^2^		**0.005**		0.19		
Static hold	Baseline	10.1	8.3–12.3	9.4	7.5–11.7	0.54
	After	10.6	9.0–14.7	10.0	8.0–13.7	0.58
	1-h FU	10.7	9.1–14.1	11.1	8.6–12	0.57
*p*-value ^2^		0.12		0.55		
Rest tone (last)	Baseline	4.0	3.1–5.4	2.9	2.4–4.8	0.15
	After	3.5	2.8–4.5	3.2	2.5–4.2	0.45
	1-h FU	2.9	2.4–4.8	3.3	2.5–3.6	0.31
*p*-value ^2^		0.72		0.89		

^1^ U Mann–Whitney test; ^2^ Friedman test (main effect); sEMG: surface electromyography; PFM: pelvic floor muscle; µV: microvolt; HIES: high-inductive electromagnetic stimulation; Me: median; Q1: first quartile; Q3: third quartile; FU: follow-up.
